# Differences in Cerebral Structure Associated With Depressive Symptoms in the Elderly With Alzheimer’s Disease

**DOI:** 10.3389/fnagi.2020.00107

**Published:** 2020-05-12

**Authors:** Yue Wu, Xingqi Wu, Qiang Wei, Kai Wang, Yanghua Tian

**Affiliations:** ^1^Department of Neurology, The First Affiliated Hospital of Anhui Medical University, Hefei, China; ^2^Anhui Province Key Laboratory of Cognition and Neuropsychiatric Disorders, Hefei, China; ^3^Collaborative Innovation Center of Neuropsychiatric Disorders and Mental Health, Hefei, China; ^4^Department of Medical Psychology, Chaohu Clinical Medical College, Anhui Medical University, Hefei, China

**Keywords:** Alzheimer’s disease, depressive symptoms, voxel-based morphometry, surface-based morphometry, linear support vector machine

## Abstract

**Background**: Alzheimer’s disease (AD) is characterized by global deterioration in multiple cognitive domains. In addition to cognitive impairment, depressive symptoms are common issues that trouble AD patients. The neuroanatomical basis of depressive symptoms in AD patients has yet to be elucidated.

**Method**: Twenty AD patients and 22 healthy controls (HCs) were recruited for the present study. Depressive symptoms in AD patients and HCs were assessed according to the Hamilton Depression Rating Scale (HDRS). Anatomical structural differences were assessed between AD patients and HCs using voxel-based morphometry (VBM) and surface-based morphometry (SBM). Correlation analyses were conducted to investigate relationships between depressive symptoms and structural altered regions. Multiple pattern analysis using linear support vector machine (SVM) was performed in another independent cohort, which was collected from Alzheimer’s Disease Neuroimaging Initiative (ADNI) data and contained 20 AD patients and 20 HCs, to distinguish AD patients from HCs.

**Results**: Compared with HCs, AD patients exhibited global cerebral atrophy in gray matter volume (GMV) and cortical thickness, including frontal, parietal, temporal, occipital, and insular lobes. In addition, insular GMV was negatively correlated with depressive symptoms. Moreover, SVM-based classification achieved an accuracy of 77.5%, a sensitivity of 70%, and a specificity of 85% by leave-one-out cross-validation.

**Conclusion**: GMV of the insula displayed atrophy among AD patients, which is associated with depressive symptoms. Our observations provide a potential neural substrate for analysis to examine the co-occurrence of AD with depressive symptoms.

## Introduction

Alzheimer’s disease (AD) is considered as a global public health problem by the World Health Organization (Lane et al., 2018). AD, which was first identified by Alois Alzheimer, is characterized by global deterioration in multiple cognitive domains (Petersen, 2004; Rémy et al., 2005; Chi et al., 2014; Chandra et al., 2019). It is the most common cause of dementia worldwide (Ohnishi et al., 2001; Nho et al., 2012; Wachinger et al., 2016; Mrdjen et al., 2019).

In addition to the progressive impairment in cognitive areas, neuropsychiatric symptoms (NPS) and behavioral issues are common concerns for AD patients (Engedal et al., 2011; Benoit et al., 2012; Knapskog et al., 2014). Four neuropsychiatric sub-symptoms have been proposed in AD: psychosis (delusion, hallucination, and sleep disorder), affective symptoms (depression and anxiety), apathy (apathy and appetite disorder), and hyperactivity (Lozupone et al., 2018). Approximately half of AD patients suffer from a depressive episode at least once during the clinical course (Lyketsos and Olin, 2002; Starkstein et al., 2005). AD accompanied by depressive symptoms is associated with great social, medical, and economic burdens.

The use of magnetic resonance imaging (MRI) for the observation of brain morphometry has been applied by numerous researchers as the resolution of an anatomical scan of a whole brain increases and acquisition times decrease (Matsuda, 2013, 2016). MRI is regarded as an effective method to ascertain the stage of the disease and to assess progression in AD (Frisoni et al., 2007). Voxel-based morphometry (VBM) and surface-based morphometry (SBM), which are not biased to one particular structure and provide an even-handed and comprehensive assessment of anatomical differences throughout the brain, provide valid methods to observe the abnormal altered brain structures in the disease (Luders et al., 2004). Previous studies revealed brain structure alterations among AD patients; such studies have made an important impact on our understanding of the disease progress.

The neuroanatomical correlates of affective symptoms in AD patients have yet to be elucidated, and it is still unclear whether such neuroanatomical changes could help to distinguish AD patients from the healthy elderly. In the present study, we aimed to observe the relationship between neuroanatomical structures and affective symptoms. We hypothesized that: (1) gray matter would be globally atrophied among AD patients; (2) depressive symptoms are believed to be caused by structural atrophy, so depressive symptoms would be correlated with such atrophy in structures; and (3) gray matter volume (GMV) in these depressive symptom-associated regions would allow for the classification of AD patients and the healthy elderly. We collected 3D T1-weighted anatomic images from 20 AD patients and 22 matched healthy controls (HCs). We used VBM and SBM to assess regions of potential differences in cortical volume and thickness. Then, we tested for correlations between volume or thickness and affective symptoms, as evaluated just by the Hamilton Depression Rating Scale (HDRS). Finally, a multiple pattern analysis using a linear support vector machine (SVM) was employed to test whether these altered neural indices are useful biomarkers for diagnosing AD in a 20-20 dataset obtained from Alzheimer’s Disease Neuroimaging Initiative (ADNI).

## Materials and Methods

### Participants

Twenty AD patients were recruited from the First Affiliated Hospital of Anhui Medical University in Anhui province, China. The AD subjects were clinically diagnosed by a specialist in accordance with the NINCDS-ADRDA (Dubois et al., 2007) criteria: (a) meeting the criteria of possible or probable AD; (b) having a Mini-Mental State Examination (MMSE) score of <24; and (c) having a Clinical Dementia Rating (CDR) score ranging from 0.5 to 2. Exclusion criteria for this study were substance use disorder, other neurological disorders, and life-threatening somatic disease.

Twenty-two matched HCs were included in this study. HCs were recruited from the local community through advertisement or were the spouses of the study patients. HCs fulfilled the following criteria: cognitively normal, no neurological or psychiatric disorders, no psychoactive medication use, an MMSE score of 28 or higher, and a CDR score of 0.

All participants were right-handed and provided written informed consent. The study was in accordance with the latest revision of the Declaration of Helsinki, and the experimental procedures were approved by the local ethics committees of Anhui Medical University.

### Neuropsychological Assessment

All patients and controls underwent a clinical evaluation and neuropsychological assessment. The following neuropsychological tests were administered to each subject to establish a clinical diagnosis as described previously (Woodward et al., 2017). (i) General cognitive functions were assessed using the MMSE test (Folstein et al., 1975). (ii) The CDR was used as a proxy of disease severity (Morris, 1993). (iii) Daily function was assessed using the Lawton–Brody Activities of Daily Living (ADL) scale (Salmon and Bondi, 2009). (iv) The Hamilton Anxiety Scale (HAMA) and the HDRS were used as evaluation for affective symptoms. Testing was administered by board-certified neuropsychologists and research staff. The MMSE test was carried out in a face-to-face interview. Neuropsychologists or research staff posed the items of the MMSE questionnaire to participants, which was used to evaluate the orientation, attention, calculational function, memory, and language of patients. According to the patients’ performance on these items, we determined an MMSE score. The ADL scale was evaluated in a similar manner. The CDR and ADL scores were obtained for each patient. Both the HAMA and HDRS were evaluated by two different research staff members dependently. Then the average scores were regarded as the anxious and depressive scores.

### MRI Data Acquisition

3D T1-weighted anatomic MRI (structural MRI, sMRI) images for each participant were obtained using a 3-T scanner (Signa HDxt 3.0T, General Electric HD 750 w, Buckinghamshire, UK) at the First Affiliated Hospital of Anhui Medical University. The T1-weighted images were acquired using a brain volume sequence with the following parameters: repetition time = 8.676 ms, echo time ratio = 3.184 ms, flip angle = 8°, field of view = 256 × 256 mm^2^, matrix size = 256 × 256, slice thickness = 1 mm, voxel size = 1 × 1 × 1 mm^3^, and number of sections = 188.

### VBM Analysis

VBM analyses were performed to determine potential differences in GMV between the AD group and the HC group. T1-weighted anatomic images were preprocessed using the VBM8 toolbox^1^ in SPM8 (Statistical Parametric Mapping software)^2^. Each structural image was segmented into gray matter, white matter, and cerebrospinal fluid using a fully automated algorithm within SPM8 and subsequently transformed to the Montreal Neurological Institute (MNI) space using diffeomorphic anatomical registration through exponentiated Lie algebra (DARTEL) normalization. Next, the normalized gray matter images were smoothed (FWHM = 8 mm) for statistical analyses. Finally, an independent-samples *t*-test, with whole-brain volume as covariant, was conducted on these normalized gray matter images to determine structural differences. Voxel-wise false discovery rate (FDR) correction was used for multicomparison correction to control type I error (*p* < 0.01, FDR corrected, minimum cluster size > 100 voxels). Significant regions were saved as masks for further analysis. Statistical analysis for GMV and saving masks were completed by the Data Processing and Analysis of Brain Imaging toolbox (DPABI)^3^.

### SBM Analysis

SBM analyses were performed to test for cortical differences between the AD group and the HC group. The T1-weighted anatomic images were processed with the CAT12 toolbox (Computational Anatomy Toolbox)^4^, which runs within SPM12 (Statistical Parametric Mapping software)^2^. Each image was processed with the following steps: segmentation, central surface, and cortical thickness estimation; topical correction; spherical mapping; and registration. Then cortical thickness parameters based on the Desikan–Killiany atlas were extracted for region-of-interest (ROI) analysis (Gaser and Dahnke, 2016). Finally, an independent-samples *t*-test was conducted to evaluate the relationship between cortical thickness and attentional effects in two groups. FDR was used for multiple comparison correction to control type I error (*p* < 0.01, FDR corrected). Cortical thickness of cerebral regions showing significant results was extracted for further analysis.

### Correlation Analysis

We performed a Pearson correlation analysis between each subject’s GMV and HDRS and between each subject’s cortical thickness and HDRS in significant regions to further explore whether neuroimaging indices were related to depressive symptoms. To observe the correlation between cortical thickness and depressive symptoms, we extracted the cortical thickness values of significant regions and performed a correlation analysis between them and HDRS. Similarly, to test for a correlation between GMV and HDRS, we performed a correlation between GMV and scales in cerebral regions showing significant atrophy. This step was completed with DPABI and masks saved in “VBM Analysis” section. Correlation analyses between GMV or cortical thickness and neuropsychological assessment, such as ADL, MMSE, and CDR, were also conducted to rule out the possibility that the atrophy of the brain region related to HDRS could be also associated with a more severe cognitive impairment. It is worth mentioning that the CDR score was logarithmically (lnCDR) converted for Gaussian distribution. The significance level was set at *p* < 0.05.

### Multivariate Pattern Analysis Using SVM

To test whether identified neural indices might serve as biomarkers for classifying AD patients from HCs, a linear SVM approach within a library for SVMs (LIBSVMs) toolkit running on MATLAB (Chang and Lin, 2011) was performed. The volume or cortical thickness showing significant correlation to affective symptoms, together with volume showing significant differences between two groups, was used as the feature for classification. We downloaded another 20 3D T1-weighted anatomic images of AD patients and another 20 3D T1-weighted anatomic images of age-/sex-matched HCs from ADNI for classification. The leave-one-out cross-validation strategy was conducted, and the performance of a classifier was assessed using the classification accuracy, sensitivity, and specificity based on the results of the cross-validation.

## Results

### Demographic and Clinical Characteristics

There were no significant differences in either age or sex between the AD and HC groups. As expected, HDRS, HAMA, MMSE, CDR, and ADL differed markedly, with significantly worse performance among the AD group compared to that among HCs. The results are shown in Table 1.

**Table 1:** Demographic information.

	AD [M (SD)/M (IQR)]^△^	HC [M (SD)/M (IQR)]^△^	χ^2/*t*/*Z*^	*p*-value
Gender (male/female)	11/9	13/9	0.07^a^	0.79
Age (years)	67.15 (11.46)	68.41 (7.19)	0.43^b^	0.67
MMSE	15.55 (3.99)	28.40 (1.53)	13.53^b^	<0.001**
HAMA	6.15 (3.39)	3.14 (2.47)	3.31^b^	0.002*
HDRS	5.05 (3.78)	2.00 (2.78)	3.00^b^	0.005*
ADL	31.45 (8.80)	20.27 (0.63)	5.66^c^	<0.001**
CDR	1.15 (0.54)	0.02 (0.11)	9.17^c^	<0.001**

^△^Normally distributed data are described as mean [standard deviation; M (SD)], and non-normally distributed data are described as median (interquartile range) [M/(IQR)]. ^a^Pearson chi-squared test; ^b^two-sample *t*-test; ^c^Mann–Whitney test; *significant at the 0.01 level (two-tailed); **significant at the 0.001 level (two-tailed). MMSE, Mini-Mental State Examination; HAMA, Hamilton Anxiety Scale; HDRS, Hamilton Depression Rating Scale; ADL, Activities of Daily Living scale; CDR, Clinical Dementia Rating Scale.

### GMV Changes and Correlation Results

**Figure 1:**
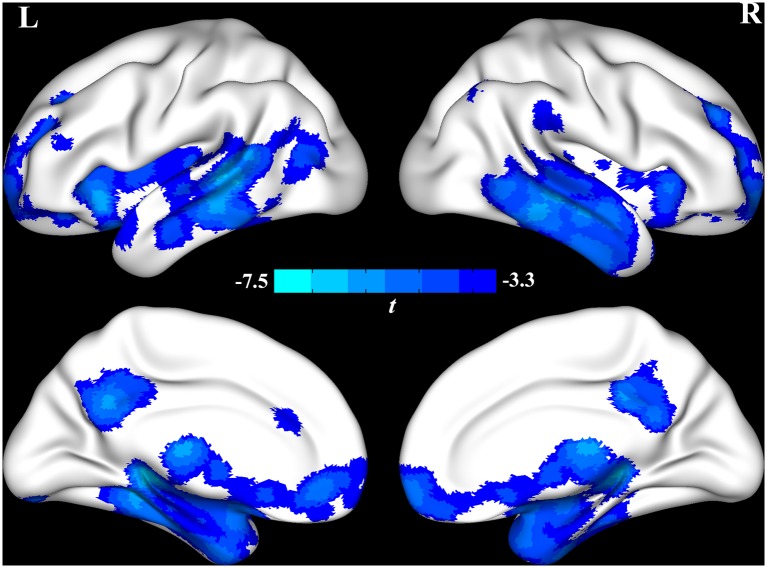
The voxel-based morphometry (VBM) analysis identified that global cerebral regions, including frontal, parietal, insular, temporal, and occipital lobes and subcortical regions, were atrophied in the Alzheimer’s disease (AD) group.

**Figure 2:**
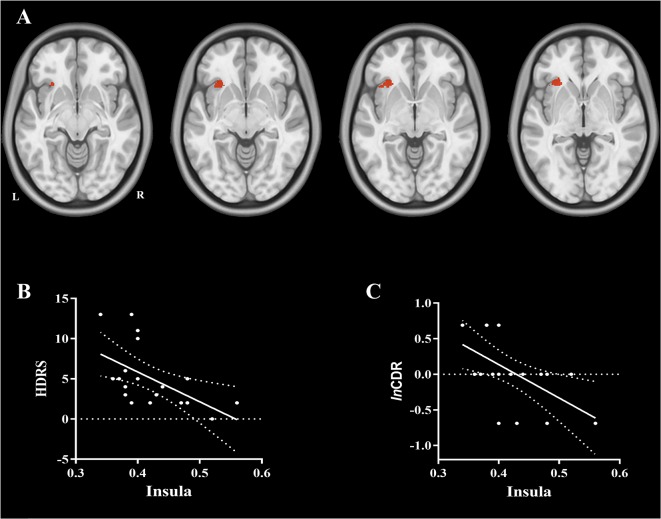
**(A)** The brain region of the insula showing a significant negative correlation with the score of hamilton depression rating scale (HDRS) and logarithmic Clinical Dementia Rating (lnCDR). **(B)** A significant negative correlation was observed between gray matter volume (GMV) of the insula and the score of HDRS (*r* = −0.53 and *p* = 0.015, respectively), which implied that the more the atrophy for the insula, the more severe is the depressive symptom. **(C)** A significant negative correlation was observed between GMV of the insula and the lnCDR (*r* = −0.55 and *p* = 0.012), which implied that the more atrophy for the insula, the more severe is the cognitive impairment.

The VBM analysis identified that global cerebral regions, including frontal, parietal, insular, temporal, and occipital lobes and subcortical regions displayed more atrophy in the AD group (see Figure 1 and Supplementary Table S1). In the correlation analysis, the GMV of insula displayed a significant negative correlation with the score of HDRS (*r* = −0.53 and *p* = 0.015, see Figure 2), which implied further atrophy of the insula, and this atrophy was more severe for the participants with depressive symptoms. The correlation analysis between the insula and other neuropsychological assessments was performed to rule out the possibility that the atrophy of the insula could be also associated with a more severe cognitive impairment. There was no significant correlation between GMV of the insula and MMSE or ADL (*r* = 0.33 and *p* = 0.15 for MMSE and *r* = −0.31 and *p* = 0.19 for ADL). However, a significant negative correlation between GMV of the insula and the score of lnCDR (*r* = −0.55 and *p* = 0.012) was observed. To exclude the effect of CDR on the correlation between the insula and HDRS, a partial correlation between GMV of the insula and HDRS with lnCDR as covariate was conducted. The result indicated a significant negative correlation (*r* = −0.50 and *p* = 0.048). We also performed a correlation analysis between atrophy GMV and ADL, MMSE, or lnCDR (see Supplementary Table S2, Figures S1–S3).

### Cortical Thickness Changes and Correlation Results

**Figure 3:**
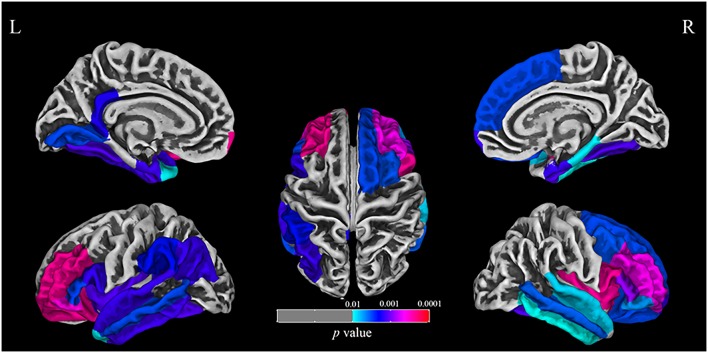
The surface-based morphometry (SBM) analysis identified that the cortex including frontal, temporal, insular, and occipital lobes was thinner in the AD group.

The SBM analysis identified that the cortex in regions including frontal, temporal, insular, and occipital lobes was thinner in the AD group (see Figure 3 and Supplementary Table S3). No significant correlation was observed between cortical thickness and affective symptoms. We also performed a correlation analysis between atrophy cortical thickness and ADL, MMSE, or lnCDR (see Supplementary Tables S4, S5).

### Classification Results

**Figure 4:**
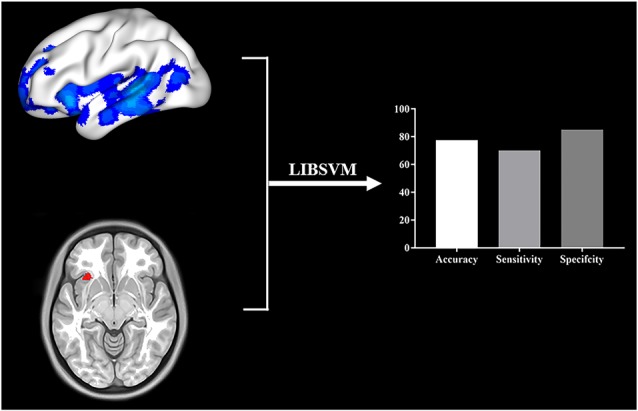
Multivariate pattern analysis using LIBSVM was applied to provide provisional evidence to determine whether identified neural indices might serve as biomarkers for diagnosing AD. The GMV of brain regions showing difference in VBM analysis and the GMV of brain regions significantly correlated to the HDRS were used as features for classification. We used a leave-one-out cross-validation strategy to estimate the generalization ability of our classifier. The classification accuracy, sensitivity, and specificity were shown.

Using the combined features of the GMV of brain regions that displayed differences in the VBM analysis and the GMV of brain regions significantly correlated to the HDRS, the linear SVM classifier achieved an accuracy of 77.5%, a sensitivity of 70%, and a specificity of 5% (Figure 4).

## Discussion

The aim of this study was to explore the probable neuroanatomical substrate of depressive symptoms of AD patients. VBM and SBM analyses indicated global deterioration in cerebral regions including the frontal, temporal, parietal, and insular areas. The degeneration of volume in the insula and inferior frontal lobe was associated with depressive symptoms among AD patients. Pattern analysis using linear SVM further demonstrated that these indices could be regarded as an auxiliary biomarker for diagnosing AD. These observations imply that the insula and inferior frontal lobe are crucial in AD progression.

In this study, we used VBM to analyze the atrophic differences between AD patients and HCs and observed the correlation between brain structures and depressive symptoms, which avoided the bias that may arise from choosing ROIs or from traditional manual structural analysis (Luders et al., 2004). The process for VBM in this study was easily operated in the SPM, which may be conveniently used in future studies. Moreover, identifying such neuroanatomical substrates for the purposes of classifying AD patients from the healthy elderly was conducted in another cohort, the ADNI cohort, which increases the reliability of our findings.

We also observed global cerebral atrophy among AD patients, which is consistent with previous structural studies (Ohnishi et al., 2001; Kim et al., 2016). We observed that depressive symptoms were associated with the insular atrophy in AD patients. Although the cognitive impairment was also associated with the insular atrophy, a significant correlation between the insula and depressive symptoms was observed after excluding the effect of the cognitive impairment. The result strongly indicated that the insula is vital in the modulation of emotion, which is consistent with previous research that demonstrated insular structures to be abnormal in patients with depressive disorder (Hayata et al., 2015; Ambrosi et al., 2017; Cooper et al., 2019; Whitton et al., 2019; Xu et al., 2019). The insula, as a vital visceral sensory node, is significantly correlated with somatic symptoms in depressed patients (Zu et al., 2019). This implies that depressive symptoms in AD patients may be associated with abnormal somatic sensation which is modulated by the insula. There is some discrepancy between our results and some studies that report depressive symptoms associated with the temporal lobe (Lebedev et al., 2014; Lebedeva et al., 2014). This might be caused by the different courses of disease for patients in these studies. As the disease progresses, patients tend to be more apathetic and may display fewer affective symptoms. Different regions may influence NPS in different courses of a disease. To the best of our knowledge, AD, which is the most common cause of dementia, involves multiple cognitive impairments (Ohnishi et al., 2001; Nho et al., 2012; Wachinger et al., 2016; Mrdjen et al., 2019). Furthermore, our results also demonstrate that the atrophy in the insula may be regarded as a biomarker for distinguishing AD patients from the healthy elderly. This observation is consistent with previous research indicating that insular atrophy complicates cognitive functions (Gasquoine, 2014; Rolls, 2016; Krohne et al., 2019).

There are some limitations to the present study. First, the number of enrolled patients with AD is small. Larger samples are needed to further validate this finding. Given the small sample size in this study, we performed the pattern analysis using linear SVM in another cohort collected from ADNI. Second, the control group lacked depressed non-AD patients. Third, to date, the NINCDS-ADRDA is considered quite obsolete because of their low specificity, which could limit the generalizability of the results. Fourth, some previous research indicates that white matter is associated with affective symptoms in AD patients (Charlton et al., 2014; Reppermund et al., 2014; Wang et al., 2014), methods used in analyzing white matter, that is, TBSS, should be applied in future studies.

## Data Availability Statement

The raw data supporting the conclusions of this article will be made available by the authors, without undue reservation, to any qualified researcher.

## Ethics Statement

The studies involving human participants were reviewed and approved by the Research Ethics Committee of the First Affiliated Hospital of Anhui Medical University. The patients/participants provided their written informed consent to participate in this study.

## Author Contributions

YW analyzed the data and wrote the manuscript. XW collected the demographic data and assessed the scales. QW, KW, and YT designed the experiment.

## Conflict of Interest

The authors declare that the research was conducted in the absence of any commercial or financial relationships that could be construed as a potential conflict of interest.

## Footnotes

^1^http://dbm.neuro.uni-jena.de/vbm.html

^2^http://www.fil.ion.ucl.ac.uk/spm

^3^http://rfmri.org/dpabi

^4^http://dbm.neuro.uni-jena.de/cat12/
