# Supplementary material for: Differences in Cerebral Structure Associated With Depressive Symptoms in the Elderly With Alzheimer’s Disease
Source: Front Aging Neurosci. 2020 May 12;12:107. doi: 10.3389/fnagi.2020.00107 (PMC7236549; doi:10.3389/fnagi.2020.00107)
Supplement: Supplementary file 1 [file Table_1.DOCX]

Table S1. Brain Regions of the Atrophy GMV

|  | Cluster  Size | Hemisphere | Brain Regions  (AAL) | MNI coordinate  (x, y, z) | *t* | *p* _FDR-Corr_ |
| --- | --- | --- | --- | --- | --- | --- |
| 1 | 87923 | Right | Thalamus | 14, -15, 4 | 7.53 | < .001 |
|  |  | Left | ParaHippocampus | -27, -42, -5 | 7.47 | < .001 |
|  |  | Right | Temporal_Mid | 56, -31, -11 | 6.92 | <.001 |
| 2 | 6191 | Right | Cingulum_Mid | 5, -39, 36 | 5.74 | < .001 |
|  |  | Left | Cingulum_Post | -6, -52, 31 | 5.69 | < .001 |
|  |  | Right | Precuneus | 9, -51, 25 | 5.08 | < .001 |
| 3 | 321 | Left | Fusiform | -23, -85, -11 | 4.89 | .001 |
| 4 | 895 | Right | Angular | 38, -66, 45 | 4.21 | .001 |
|  |  | Right | Occipital_Sup | 24, -67, 48 | 3.78 | .003 |
| 5 | 497 | Left | Cingulum_Ant | -5, 29, 24 | 4.06 | .002 |
|  |  | Right | Cingulum_Ant | 3, 30, 28 | 3.67 | .003 |
|  |  | Left | Cingulum_Mid | -5, 15, 36 | 3.25 | .007 |
| 6 | 310 | Left | Parietal_Inf | -35, -60, 46 | 3.96 | .002 |
| 7 | 144 | Right | Frontal_Inf_Oper | 42, 9, 33 | 3.95 | .002 |
|  |  | Right | Frontal_Inf_Tri | 45, 21, 30 | 3.26 | .007 |
| 8 | 119 | Left | Parietal_Inf | -57, -30, 49 | 3.80 | .003 |
| 9 | 270 | Left | Precentral | -44, 8, 36 | 3.79 | .003 |
| 10 | 441 | Left | Occipital_Sup | -24, -87, 27 | 3.75 | .003 |
|  |  | Left | Occipital_Mid | -35, -75, 22 | 3.36 | .006 |
| 11 | 144 | Right | Frontal_Sup_Medial | 11, 53, 27 | 3.75 | .003 |
|  |  | Right | Frontal_Sup | 18, 56, 25 | 3.26 | .007 |
| 12 | 135 | Right | Occipital_Mid | 30, -79, 30 | 3.61 | .004 |

Abbreviations: AAL, Anatomical Automatic Labeling Atlas.

Table S2 Correlation between insular GMV, HDRS and CDR in AD patients

|  |  | *r* | *p* |
| --- | --- | --- | --- |
| Insular GMV | HDRS | -.53 | .015 |
| Insular GMV | *ln*CDR | -.55 | .012 |
| Insular GMV | HDRS (with *ln*CDR as covariance) | -.50 | .048 |

Table S3. Brain Regions of the Atrophy Thickness (Desikan-Killiany atlas) ^a^

|  | AD  (Mean (SD)) | HC  (Mean (SD)) | *t* | *p* _uncorrected_ |
| --- | --- | --- | --- | --- |
| **Left Hemisphere** |  |  |  |  |
| supramarginal | 2.51 (0.15) | 2.66 (0.11) | 3.57 | .001 |
| inferiorparietal | 2.46 (0.17) | 2.62 (0.08) | 3.82 | < .001 |
| superiortemporal | 2.72 (0.19) | 2.92 (0.15) | 3.87 | < .001 |
| rostralmiddlefrontal | 2.53 (0.13) | 2.73 (0.12) | 5.01 | < .001 |
| insula | 3.32 (0.29) | 3.62 (0.20) | 4.04 | < .001 |
| fusiform | 2.80 (0.21) | 3.00 (0.10) | 3.88 | < .001 |
| middletemporal | 2.81 (0.29) | 3.05 (0.16) | 3.39 | .002 |
| inferiortemporal | 2.84 (0.23) | 3.08 (0.16) | 4.06 | < .001 |
| lingual | 2.13 (0.11) | 2.23 (0.10) | 3.33 | .002 |
| lateralorbitofrontal | 3.06 (0.14) | 3.26 (0.12) | 4.93 | < .001 |
| parsopercularis | 2.76 (0.12) | 2.90 (0.12) | 3.83 | < .001 |
| isthmuscingulate | 2.48 (0.19) | 2.72 (0.20) | 3.94 | < .001 |
| parstriangularis | 2.66 (0.16) | 2.81 (0.12) | 3.38 | .002 |
| entorhinal | 3.52 (0.73) | 4.25 (0.45) | 3.94 | < .001 |
| parsorbitalis | 2.79 (0.15) | 2.99 (0.13) | 4.40 | < .001 |
| temporalpole | 3.80 (0.55) | 4.14 (0.18) | 2.62 | .015 |
| **Right Hemisphere** |  |  |  |  |
| superiorfrontal | 2.92 (0.14) | 3.05 (0.11) | 3.50 | .001 |
| rostralmiddlefrontal | 2.51 (0.14) | 2.71 (0.12) | 4.68 | < .001 |
| superiortemporal | 2.87 (0.19) | 3.04 (0.18) | 3.18 | .003 |
| middletemporal | 2.94 (0.20) | 3.14 (0.17) | 3.49 | .001 |
| insula | 3.43 (0.24) | 3.78 (0.22) | 4.89 | < .001 |
| fusiform | 2.92 (0.20) | 3.13 (0.14) | 3.99 | < .001 |
| lateralorbitofrontal | 3.01 (0.14) | 3.19 (0.20) | 3.42 | .002 |
| inferiortemporal | 3.04 (0.24) | 3.22 (0.16) | 2.97 | .006 |
| caudalmiddlefrontal | 2.69 (0.19) | 2.86 (0.13) | 3.50 | .001 |
| parsopercularis | 2.79 (0.13) | 2.97 (0.10) | 5.01 | < .001 |
| parstriangularis | 2.69 (0.15) | 2.88 (0.12) | 4.39 | < .001 |
| parahippocampal | 2.58 (0.31) | 2.80 (0.18) | 2.79 | .009 |
| parsorbitalis | 2.86 (0.18) | 3.04 (0.17) | 3.28 | .002 |
| entorhinal | 3.74 (0.75) | 4.46 (0.39) | 3.85 | .001 |

a: Mean cortical thickness showing significantly different in Desikan-Killiany atlas between groups were extracted for further analysis

Table S4 Matrix of *r* value for correlation between atrophy cortical thickness and MMSE, ADL, HDRS and *ln*CDR

|  | MMSE | ADL | HDRS | *ln*CDR |
| --- | --- | --- | --- | --- |
| **Left Hemisphere** |  |  |  |  |
| supramarginal | 0.07 | -0.16 | -0.18 | -0.51 |
| inferiorparietal | -0.11 | -0.07 | -0.01 | -0.18 |
| superiortemporal | 0.31 | -0.46 | 0.09 | -0.59 |
| rostralmiddlefrontal | 0.07 | -0.27 | 0.07 | -0.36 |
| insula | 0.21 | -0.51 | 0.02 | -0.48 |
| fusiform | 0.30 | -0.18 | 0.12 | -0.38 |
| middletemporal | 0.21 | -0.20 | -0.03 | -0.36 |
| inferiortemporal | 0.16 | -0.25 | -0.07 | -0.43 |
| lingual | 0.27 | -0.43 | 0.39 | -0.32 |
| lateralorbitofrontal | -0.08 | 0.15 | 0.25 | 0.04 |
| parsopercularis | -0.10 | -0.03 | -0.34 | -0.28 |
| isthmuscingulate | 0.06 | -0.19 | -0.35 | -0.25 |
| parstriangularis | 0.23 | -0.15 | -0.34 | -0.47 |
| entorhinal | 0.32 | -0.14 | 0.09 | -0.37 |
| parsorbitalis | 0.08 | -0.21 | 0.02 | -0.25 |
| temporalpole | 0.20 | -0.32 | 0.17 | -0.25 |
| **Right Hemisphere** |  |  |  |  |
| superiorfrontal | -0.33 | -0.04 | 0.08 | -0.13 |
| rostralmiddlefrontal | -0.09 | -0.46 | -0.19 | -0.35 |
| superiortemporal | 0.38 | -0.35 | 0.08 | -0.54 |
| middletemporal | 0.26 | -0.39 | -0.14 | -0.49 |
| insula | -0.01 | -0.19 | -0.12 | -0.13 |
| fusiform | 0.03 | -0.22 | -0.05 | -0.30 |
| lateralorbitofrontal | -0.02 | -0.05 | 0.04 | -0.02 |
| inferiortemporal | 0.22 | -0.29 | -0.04 | -0.36 |
| caudalmiddlefrontal | -0.24 | -0.28 | -0.04 | -0.21 |
| parsopercularis | -0.28 | -0.23 | -0.21 | -0.08 |
| parstriangularis | 0.04 | -0.12 | -0.19 | -0.27 |
| parahippocampal | 0.49 | -0.53 | 0.01 | -0.55 |
| parsorbitalis | 0.14 | -0.20 | -0.10 | -0.30 |
| entorhinal | 0.34 | -0.35 | 0.23 | -0.37 |

Table S5 Matrix of *p* value for correlation between atrophy cortical thickness and MMSE, ADL, HDRS and *ln*CDR

|  | MMSE | ADL | HDRS | *ln*CDR |
| --- | --- | --- | --- | --- |
| **Left Hemisphere** |  |  |  |  |
| supramarginal | 0.761 | 0.495 | 0.445 | 0.021 ^*^ |
| inferiorparietal | 0.649 | 0.756 | 0.953 | 0.458 |
| superiortemporal | 0.178 | 0.044 ^*^ | 0.698 | 0.006 ^**^ |
| rostralmiddlefrontal | 0.775 | 0.250 | 0.758 | 0.117 |
| insula | 0.383 | 0.020 ^*^ | 0.924 | 0.030 ^*^ |
| fusiform | 0.193 | 0.443 | 0.606 | 0.097 |
| middletemporal | 0.371 | 0.390 | 0.895 | 0.119 |
| inferiortemporal | 0.500 | 0.282 | 0.762 | 0.059 |
| lingual | 0.252 | 0.061 | 0.089 | 0.174 |
| lateralorbitofrontal | 0.723 | 0.533 | 0.284 | 0.877 |
| parsopercularis | 0.662 | 0.910 | 0.142 | 0.225 |
| isthmuscingulate | 0.802 | 0.432 | 0.130 | 0.291 |
| parstriangularis | 0.329 | 0.533 | 0.138 | 0.038 ^*^ |
| entorhinal | 0.163 | 0.542 | 0.703 | 0.104 |
| parsorbitalis | 0.724 | 0.377 | 0.948 | 0.281 |
| temporalpole | 0.400 | 0.172 | 0.465 | 0.283 |
| **Right Hemisphere** |  |  |  |  |
| superiorfrontal | 0.158 | 0.861 | 0.731 | 0.577 |
| rostralmiddlefrontal | 0.706 | 0.043 ^*^ | 0.415 | 0.136 |
| superiortemporal | 0.096 | 0.128 | 0.750 | 0.013 ^*^ |
| middletemporal | 0.276 | 0.093 | 0.557 | 0.029 ^*^ |
| insula | 0.983 | 0.414 | 0.617 | 0.585 |
| fusiform | 0.886 | 0.345 | 0.836 | 0.201 |
| lateralorbitofrontal | 0.918 | 0.820 | 0.876 | 0.942 |
| inferiortemporal | 0.358 | 0.220 | 0.858 | 0.114 |
| caudalmiddlefrontal | 0.311 | 0.236 | 0.878 | 0.364 |
| parsopercularis | 0.238 | 0.338 | 0.363 | 0.724 |
| parstriangularis | 0.861 | 0.606 | 0.419 | 0.259 |
| parahippocampal | 0.027 ^*^ | 0.017 ^*^ | 0.973 | 0.013 ^*^ |
| parsorbitalis | 0.551 | 0.397 | 0.674 | 0.199 |
| entorhinal | 0.138 | 0.135 | 0.331 | 0.111 |

*: significant level at *p* < .05; **: significant level at *p* < .01


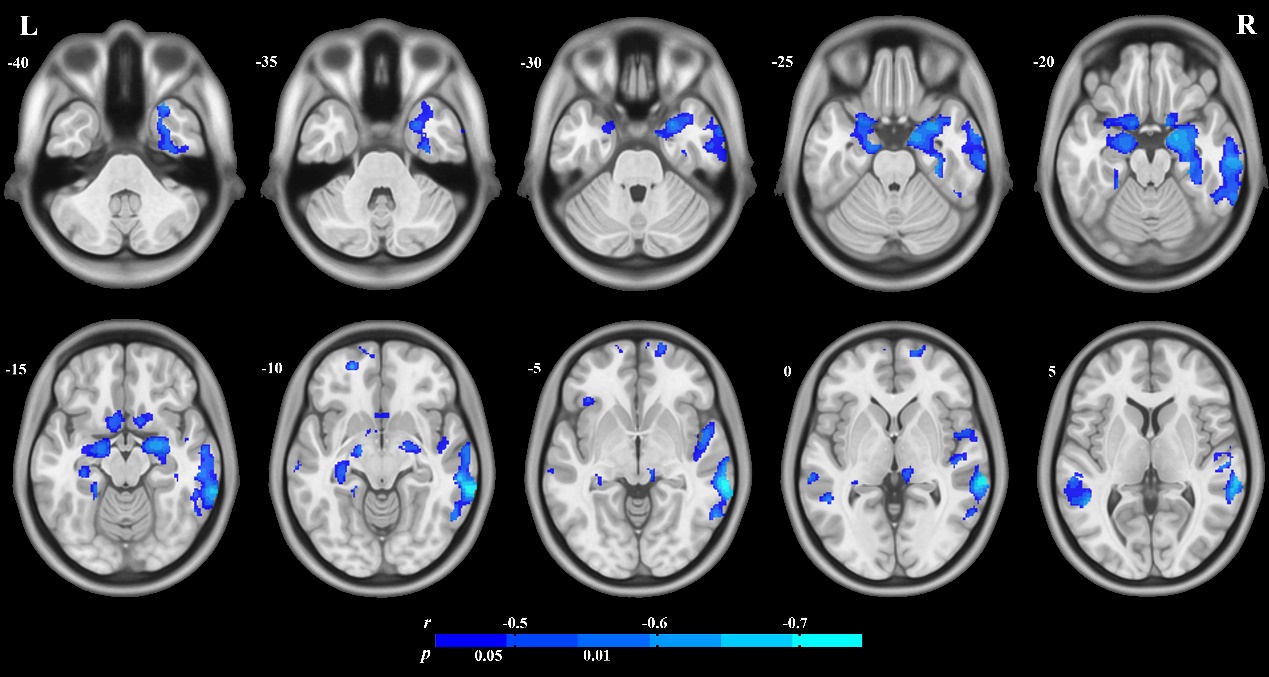


**Figure S1: Correlation between atrophy GMV and ADL:** Negative correlations between atrophy GMV and ADL score were observed, which mainly located in frontal lobe, temporal lobe and some subcortical regions such as amygdala and hippocampus. Results showed that the more atrophic in these regions, the poorer for daily ability of patients.


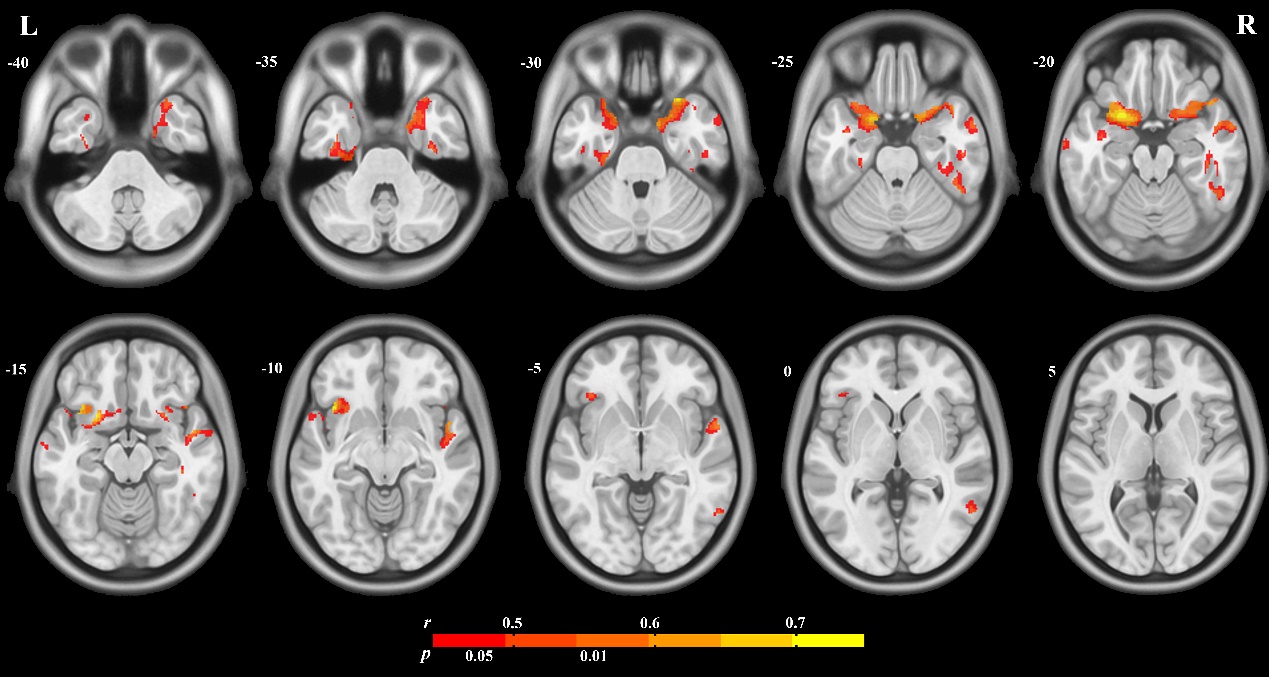


**Figure S2: Correlation between atrophy GMV and MMSE**: Positive correlations between atrophy GMV and MMSE score were observed, which mainly located in frontal lobe, temporal lobe, parietal lobe and some subcortical regions such as amygdala, hippocampus and thalamus. Results showed that the more atrophic in these regions, the more severe cognitive impairment for patients.


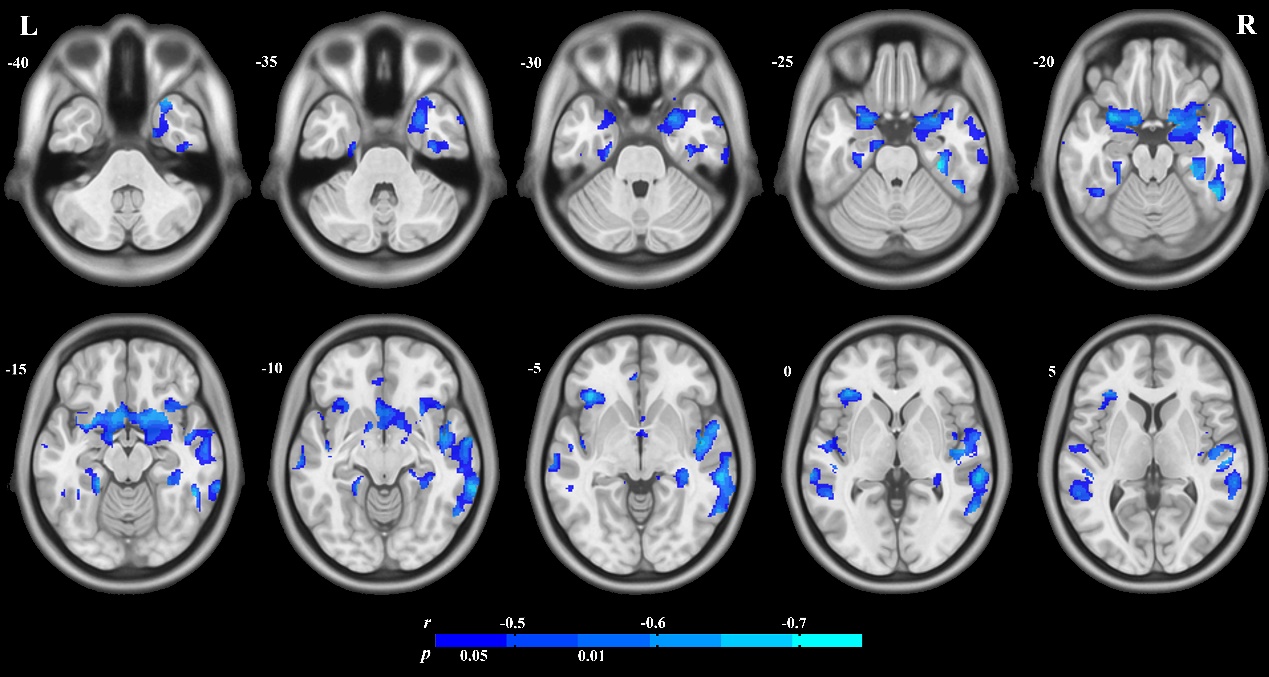


**Figure S3: Correlation between atrophy GMV and *ln*CDR:** Negative correlations between atrophy GMV and CDR score were observed, which mainly located in insula, frontal, temporal and parietal lobe, as well as some subcortical regions such as amygdala and hippocampus. Results showed that the more atrophic in these regions, the poorer for daily ability of patients.
